# Plasma biomarkers in chronic single moderate–severe traumatic brain injury

**DOI:** 10.1093/brain/awae255

**Published:** 2024-09-24

**Authors:** Gershon Spitz, Amelia J Hicks, Stuart J McDonald, Vincent Dore, Natasha Krishnadas, Terence J O’Brien, William T O’Brien, Lucy Vivash, Meng Law, Jennie L Ponsford, Christopher Rowe, Sandy R Shultz

**Affiliations:** Monash-Epworth Rehabilitation Research Centre, School of Psychological Sciences, Faculty of Medicine, Nursing and Health Sciences, Monash University, Clayton, VIC 3800, Australia; Department of Neuroscience, School of Translational Medicine, Faculty of Medicine, Nursing and Health Sciences, Monash University, Clayton, VIC 3004, Australia; Monash-Epworth Rehabilitation Research Centre, School of Psychological Sciences, Faculty of Medicine, Nursing and Health Sciences, Monash University, Clayton, VIC 3800, Australia; Department of Neuroscience, School of Translational Medicine, Faculty of Medicine, Nursing and Health Sciences, Monash University, Clayton, VIC 3004, Australia; Department of Neurology, The Alfred, Melbourne, VIC 3004, Australia; Florey Department of Neuroscience and Mental Health, University of Melbourne, Parkville, VIC 3010, Australia; Department of Molecular Imaging and Therapy, Austin Health, Heidelberg, VIC 3084, Australia; Florey Department of Neuroscience and Mental Health, University of Melbourne, Parkville, VIC 3010, Australia; Department of Molecular Imaging and Therapy, Austin Health, Heidelberg, VIC 3084, Australia; Department of Neuroscience, School of Translational Medicine, Faculty of Medicine, Nursing and Health Sciences, Monash University, Clayton, VIC 3004, Australia; Department of Neurology, The Alfred, Melbourne, VIC 3004, Australia; Department of Medicine, Royal Melbourne Hospital, The University of Melbourne, Parkville, VIC 3010, Australia; Department of Neuroscience, School of Translational Medicine, Faculty of Medicine, Nursing and Health Sciences, Monash University, Clayton, VIC 3004, Australia; Department of Neuroscience, School of Translational Medicine, Faculty of Medicine, Nursing and Health Sciences, Monash University, Clayton, VIC 3004, Australia; Department of Neurology, The Alfred, Melbourne, VIC 3004, Australia; Department of Medicine, Royal Melbourne Hospital, The University of Melbourne, Parkville, VIC 3010, Australia; Department of Neuroscience, School of Translational Medicine, Faculty of Medicine, Nursing and Health Sciences, Monash University, Clayton, VIC 3004, Australia; Department of Radiology, Alfred Health, Melbourne, VIC 3004, Australia; Monash-Epworth Rehabilitation Research Centre, School of Psychological Sciences, Faculty of Medicine, Nursing and Health Sciences, Monash University, Clayton, VIC 3800, Australia; Florey Department of Neuroscience and Mental Health, University of Melbourne, Parkville, VIC 3010, Australia; Department of Molecular Imaging and Therapy, Austin Health, Heidelberg, VIC 3084, Australia; Department of Neuroscience, School of Translational Medicine, Faculty of Medicine, Nursing and Health Sciences, Monash University, Clayton, VIC 3004, Australia; Department of Neurology, The Alfred, Melbourne, VIC 3004, Australia; The Centre for Trauma and Mental Health Research, Health Sciences and Human Services, Vancouver Island University, Nanaimo, BC V9R 5S5, Canada

**Keywords:** traumatic brain injury, chronic outcomes, blood biomarkers, MRI, PET

## Abstract

Blood biomarkers are an emerging diagnostic and prognostic tool that reflect a range of neuropathological processes following traumatic brain injury (TBI). Their effectiveness in identifying long-term neuropathological processes after TBI is unclear. Studying biomarkers in the chronic phase is vital because elevated levels in TBI might result from distinct neuropathological mechanisms during acute and chronic phases. Here, we examine plasma biomarkers in the chronic period following TBI and their association with amyloid and tau PET, white matter microarchitecture, brain age and cognition.

We recruited participants ≥40 years of age who had suffered a single moderate–severe TBI ≥10 years previously between January 2018 and March 2021. We measured plasma biomarkers using single molecule array technology [ubiquitin C-terminal hydrolase L1 (UCH-L1), neurofilament light (NfL), tau, glial fibrillary acidic protein (GFAP) and phosphorylated tau (P-tau181)]; PET tracers to measure amyloid-β (^18^F-NAV4694) and tau neurofibrillary tangles (^18^F-MK6240); MRI to assess white matter microstructure and brain age; and the Rey Auditory Verbal Learning Test to measure verbal-episodic memory.

A total of 90 post-TBI participants (73% male; mean = 58.2 years) were recruited on average 22 years (range = 10–33 years) post-injury, and 32 non-TBI control participants (66% male; mean = 57.9 years) were recruited. Plasma UCH-L1 levels were 67% higher {exp(*b*) = 1.67, *P* = 0.018, adjusted *P* = 0.044, 95% confidence interval (CI) [10% to 155%], area under the curve = 0.616} and P-tau181 were 27% higher {exp(*b*) = 1.24, *P* = 0.011, adjusted *P* = 0.044, 95% CI [5% to 46%], area under the curve = 0.632} in TBI participants compared with controls. Amyloid and tau PET were not elevated in TBI participants. Higher concentrations of plasma P-tau181, UCH-L1, GFAP and NfL were significantly associated with worse white matter microstructure but not brain age in TBI participants. For TBI participants, poorer verbal-episodic memory was associated with higher concentration of P-tau181 {short delay: *b* = −2.17, SE = 1.06, *P* = 0.043, 95% CI [−4.28, −0.07]; long delay: *b*_P-tau_ = −2.56, SE = 1.08, *P* = 0.020, 95% CI [−4.71, −0.41]}, tau {immediate memory: *b*_Tau_ = −6.22, SE = 2.47, *P* = 0.014, 95% CI [−11.14, −1.30]} and UCH-L1 {immediate memory: *b*_UCH-L1_ = −2.14, SE = 1.07, *P* = 0.048, 95% CI [−4.26, −0.01]}, but was not associated with functional outcome.

Elevated plasma markers related to neuronal damage and accumulation of phosphorylated tau suggest the presence of ongoing neuropathology in the chronic phase following a single moderate–severe TBI. Plasma biomarkers were associated with measures of microstructural brain disruption on MRI and disordered cognition, further highlighting their utility as potential objective tools to monitor evolving neuropathology post-TBI.

## Teaser

Spitz *et al.* study long-term survivors of moderate–severe TBI, and show that elevated levels of plasma biomarkers related to neuronal damage are associated with white matter microstructure disruption on MRI and disordered cognition. The results highlight the potential for blood biomarkers to track TBI-related brain changes.

## Introduction

Traumatic brain injury (TBI) is the leading cause of injury-related death and disability worldwide.^1^ There is growing interest in the long-term neuropathology and consequences of TBI, including the chronic neurological, cognitive and behavioural problems.^2-7^ In recent years, blood biomarkers have been used to investigate the evolving nature of TBI neuropathology.^8-11^ Measurement of blood biomarkers in the acute and subacute phases is, to a larger extent, affected by the direct effects of TBI. Elevated levels in the chronic phase, many years or decades after the initial injury, are likely to correspond better to ongoing neurodegenerative processes or disease.^4^ The utility of blood biomarkers has now been examined across the TBI severity spectrum.^3,10,12-27^ Neurofilament light (NfL) and tau, both of which are blood markers of axonal damage, are elevated following moderate–severe TBI and are predictive of worse outcome.^3^ However, only NfL remains elevated over the first several years following injury.^13^ Ubiquitin C-terminal hydrolase L1 (UCH-L1) and glial fibrillary acidic protein (GFAP), which are markers of neuronal and astrocytic pathology, respectively, are also elevated acutely, but largely normalize within the first year following injury.^3^

The relevance of blood biomarkers to TBI-specific neurological processes has been examined using multimodal designs that include neuroimaging with MRI and PET. Within the first 5 years following injury, higher blood concentrations of NfL, tau, GFAP and UCH-L1 are associated with MRI measures of axonal injury and brain atrophy.^3,25^ A single study has examined the abovementioned blood biomarkers in the chronic phase of moderate–severe TBI and their association with PET and MRI.^2^ In a small sample (*n* = 21) at an average of 31 years post-injury, blood plasma concentrations of NfL, tau, GFAP, UCH-L1 and CSF phosphorylated tau (P-tau) did not differ between TBI and healthy control participants, despite greater binding of PET flortaucipir (which binds to tau neurofibrillary tangles) in the occipital cortex of individuals with TBI.

Here, we investigated these blood biomarkers in a large sample (*n* = 90) of individuals with a single moderate–severe TBI in the chronic (>10 years) phase. We used a multimodal design, including a panel of plasma biomarkers that measure neuronal (UCH-L1, NfL, tau and P-tau181) and astrocytic (GFAP) pathology. We characterized plasma profiles in relationship to two PET tracers: (i) ^18^F-NAV4694, a measure of amyloid-β (Aβ); and (ii) ^18^F-MK6240, a measure of tau neurofibrillary tangles. We complemented this analysis by examining the association between plasma markers and MRI measures of white matter microstructure and brain age. Last, we investigated the relationship between plasma markers and verbal-episodic memory, which is a domain we have previously shown to be associated with neuropathology present in chronic TBI.^28,29^

## Materials and methods

### Study population

Participants were recruited to the present study between January 2018 and March 2021 from a database of individuals who had been admitted to an inpatient rehabilitation programme at Epworth HealthCare in Melbourne, Victoria, Australia. All individuals, or their legal representatives, gave written permission before any research activities, in line with the Declaration of Helsinki. The research received approval from the Austin Health Human Research Ethics Committee (HREC/17/Austin/202). From this larger database, participants were contacted for participation in a study that examined chronic neuropathology in an adult ageing cohort of individuals with moderate–severe TBI. Participants included in this study met several eligibility criteria: they were aged 40 years or older at the time of enrolment and had experienced a single moderate–severe TBI, as determined by medical records and classified according to the Mayo classification system.^30^ Participants were required to be ≥16 years old at the time of injury and to have sustained the injury ≥10 years prior to participation. Individuals were excluded if they had a pre-existing neurological condition and lacked the cognitive or functional capacity to undergo an in-person assessment, which included a blood sample, neuropsychological evaluation, MRI and PET scans. This cohort of ageing, chronic TBI participants has been described elsewhere.^28,29,31^ Importantly, we shown that this study cohort does not differ from the broader database on important variables, including sex, age at injury, injury severity [post-traumatic amnesia (PTA) and Glasgow Coma Score (GCS)] and mechanism of injury. Given the aims of the present study, in comparison to our previous studies using this ageing cohort, participants in the present study were required to have had a blood sample.

Control participants were also recruited and were required to be 40 years or older, have no history of TBI or loss of consciousness and have sufficient English language skills and cognitive capacity to participate in the study. In comparison to previous studies using our ageing cohort, we ran an initial step to ensure that control participants were matched to the TBI cohort with respect to age at assessment, sex and premorbid intelligence quotient (IQ). This was conducted using the MatchIt package in R.^32^ A detailed description of this procedure can be found in the Supplementary material, Methods 1.

Fasted blood sampling was performed at the first PET session. MRI and a clinical assessment were completed on the same day. The MRI and earliest PET scan were conducted an average of 50 days [standard deviation (SD) = 33 days] apart.

### Blood biomarker procedures

Blood biomarker procedures followed the protocol set by the Australian Imaging, Biomarkers and Lifestyle (AIBL) Study.^33^ Plasma from K_2_-EDTA tubes (7.5 ml S-monovette 01.1605.008, Sarstedt) containing pre-added prostaglandin E_1_ (33 ng/ml of whole blood, Sapphire Biosciences) to prevent platelet activation, was centrifuged at room temperature at 200*g* for 10 min, at 800*g* for 15 min, and finally, at 3200*g* for 30 min (‘max *g*’). Platelet-rich plasma was collected and combined after each centrifuge process, until ‘max *g*’ plasma was snap frozen within 2 h of collection and stored in vapour-phase liquid nitrogen. This AIBL protocol was optimized to prevent platelet activation, a potential source of peripheral Aβ. The single molecular array (Simoa) HD-X platform was used to measure GFAP, UCH-L1, NfL and total tau concentrations using the ‘Neurology 4-Plex B’ assay. P-tau181 concentration was analysed using the ‘P-tau181 Advantage V2’ assay. Samples were measured in duplicate, yielding an average coefficient of variation of 2.8% for GFAP, 5.4% for NfL, 6.2% for tau, 28.5% for UCH-L1 and 5.4% for P-tau181. Thirty-two of 147 samples measured below the lower limit of detection for UCH-L1 (1.90 pg/ml) and were therefore allocated concentrations equal to the lower limit of detection.

### MRI procedures

#### White matter microstructure

Participants completed a diffusion-weighted sequence using a Siemens Magnetom Skyra 3 T scanner (for protocol details, see Supplementary material, Methods 2). MRI diffusion processing was conducted using MRtrix3.^34^ Diffusion-weighting imaging data were denoised, Gibbs ringing artefacts were removed, and the data underwent motion, eddy current distortion and bias field correction. Susceptibility distortion was corrected using a reverse-phase diffusion-weighted imaging (DWI) image. Tissue-specific response functions for white matter, grey matter and CSF were generated using a single-shell three-tissue response function using the ‘Dhollander’ estimation approach in MRtrix3. Response functions from all participants were averaged to generate a unique cohort response function for each tissue type. Fibre orientation distributions (FODs) were reconstructed from the cohort white matter and CSF response functions using the multi-tissue constrained spherical deconvolution algorithm. Global intensity normalization was performed to correct for intensity inhomogeneities to enable quantitative group comparisons. An unbiased study-specific white matter FOD template was created using the FOD-guided non-linear registration based on the white matter FODs of 50 TBI subjects and 50 controls, including extra controls recruited from Epworth HealthCare beyond those directly involved in this study to increase template robustness. Each group consisted of an equal number of male and female participants. Each participant’s FODs was then registered to the FOD template and fixels computed by performing FOD segmentation. Fixels at the individual level were all reoriented to the corresponding fixels of the FOD template. Whole-brain probabilistic tractography was performed on the FOD template to enable connectivity-based fixel enhancement for fixel statistics. Twenty million streamlines were generated using the probabilistic iFOD2 algorithm. The total number of streamlines was then filtered down to 2 million by applying spherical deconvolution informed filtering of tractograms (SIFT) to reduce reconstruction biases. FD, measuring fixel-wise fibre density of specific white matter fibre bundles within voxels, and log-FC, measuring macrostructural change of a fibre bundle in a logarithm scale, were extracted for each participant. In addition, we calculated traditional tensor-based metrics, including fractional anisotropy (FA), apparent diffusion coefficient (ADC), axial diffusivity (AD) and radial diffusivity (RD). We used TractSeg, a deep learning-based framework for automated white matter bundle segmentation, to segment the white matter FOD template into 52 white matter fibre tracts (Supplementary Fig. 1).^35^ Average fixel and tensor metrics were calculated for each white matter bundle for each study participant.

#### Brain age

‘Brain age’ was used as a measure of brain volume change and atrophy. Our group has previously published on brain age changes in this cohort, showing brain age to be elevated in TBI subjects compared with controls.^28^ In the present manuscript, brain age estimates were derived using the brainageR (https://github.com/james-cole/brainageR) package in R, which generates a brain-predicted age value from raw T_1_-weighted MRI scans.^36^ brainageR segmented and normalized raw T_1_-weighted MRI scans. Images of segmented T_1_-weighted images were inspected visually for quality control. Normalized T_1_-weighted images were vectorized, and grey matter, white matter and CSF vectors masked. Grey matter, white matter and CSF volumes were used to predict a single predicted brain age value for each participant. Predicted brain age was initially corrected owing to a known bias, whereby brain predicted age is overestimated for younger individuals and underestimated for older individuals. To adjust for this age bias, we applied the following correction.^37^ We first fitted Y = *α* × *Ω* + *β*, where *Y* is the modelled predicted age as a function of chronological age (Ω), *α* represents the slope, and *β* is the intercept. The derived values of the slope (α) and intercept (β) were used to correct predicted brain age using the following calculation: Corrected predicted brain age = Predicted brain age + [Ω−(α×Ω+β)]. Our key measure of brain ageing was the brain age gap, which was calculated by subtracting the chronological age of each participant from their corrected predicted ‘brain age’ (Brain age gap = Corrected predicted brain age − Chronological age). Positive ‘brain age’ gap values indicate a biologically older brain, and negative brain age gap values suggest a biologically younger brain.

### PET procedures

^18^F-NAV4694 and ^18^F-MK6240 PET images were acquired over two sessions (mean gap between sessions = 13.5 days, SD = 36.6 days). Tracer labelling was performed in the Department of Molecular Imaging and Therapy, Austin Health, immediately prior to administration. The final product had a radiochemical purity of >95%. PET scans were acquired using a Phillips Gemini PET/CT scanner or a Siemens Biograph PET/CT. A CT scan was performed for attenuation correction immediately prior to each imaging period. Twenty-minute static scans were acquired 50 min after injection of 200 ± 15 MBq of ^18^F-NAV4694 and 90 min after injection of 184.5 ± 15 MBq of ^18^F-MK6240.

PET scans underwent spatial normalization using Computational Analysis of PET from AIBL (CapAIBL®) software^38^ and were scaled to the cerebellar cortex. CapAIBL is an innovative automated semi-quantitative method that enables standardized uptake value ratio quantification of PET scans without the necessity for a corresponding MR image.^38,39^ Instead, CapAIBL uses an adaptive PET template for registration of each PET image (detailed description of the CapAIBL pipeline is given by Borgeat *et al*.^39^). The standardized uptake value ratio was computed for both ^18^F-NAV4694 and ^18^F-MK6240 PET images. Aβ was quantified using the calibrated centiloid method for CapAIBL applied for quantification, using the whole cerebellum as the reference region.

^18^F-MK6240 standardized uptake value ratio values were calculated across four composite regions of interest carefully selected to mirror established patterns of tau propagation and accumulation in Alzheimer’s disease.^40^ These regions of interest correspond to recognized tau staging frameworks. The initial stage is characterized by the mesial temporal region, encompassing the entorhinal cortex, hippocampus, parahippocampus and amygdala. Progressing to the next stage, we examine the temporoparietal region, which includes the inferior and middle temporal gyri, fusiform gyrus, supramarginal gyrus, angular gyrus, posterior cingulate/precuneus, superior and inferior parietal lobules and lateral occipital cortex. The final stage of tau deposition is observed in the ‘rest of neocortex’ region of interest, covering the dorsolateral and ventrolateral prefrontal cortex, orbitofrontal cortex, gyrus rectus, superior temporal gyrus and anterior cingulate cortex. Importantly, these regions (mesial temporal, temporoparietal and rest of neocortex) are distinct and non-overlapping. Additionally, we incorporated a meta-temporal region into our analysis, aligning with emerging evidence suggesting its utility in capturing early tau accumulation. This meta-temporal region shares some anatomical overlap with mesial temporal and temporoparietal, encompassing the entorhinal cortex, hippocampus proper, parahippocampus, amygdala, fusiform gyrus, inferior and middle temporal gyri, temporo-occipital region and angular gyrus.

### Cognitive assessment

Our primary cognitive outcome of verbal-episodic memory was measured using the Rey Auditory Verbal Learning Test (RAVLT), which has been shown to be sensitive to chronic consequences of TBI and a sensitive marker of neurodegeneration.^28,41,42^ We measured immediate memory (Trials 1–5 sum), short delay (Trial 6) and long delay (Trial 7).

We included secondary cognitive measures, including the Digit Span Forwards and Backwards, Digit Symbol Coding Logical Memory I and II, Rey-Osterrieth Complex Figure (ROCF) copy score, 3, and 30 min delay, Controlled Oral Word Association Test and the Trail Making Test parts A and B.

### Statistical analysis

Statistical analyses were conducted in R, v.4.2.2, unless otherwise stated. A natural logarithmic transformation was applied to all blood markers owing to skewed distributions. A single TBI participant was excluded from analyses including P-tau181 owing to an extreme value, equivalent to a *z*-score of 11.5 compared with the rest of the group. Group differences in blood plasma concentrations were conducted using linear regression, controlling for age and sex. In these linear regressions where the outcome was the logarithm of the blood biomarker, coefficients were exponentiated. For instance, a group beta coefficient of 1.5 indicates that the blood biomarker concentrations are expected to increase by ∼50% compared with the control group. Furthermore, to control for multiple comparisons, a false discovery rate (FDR) correction was used for all group-effect *P*-values. Area under the receiver operating characteristic (AUC) curves were computed for blood biomarker differences between groups, adjusting for age and sex, using the ROCnReg package.^43^ The associations between time since injury, PTA, GCS and blood biomarker concentrations were examined using linear regressions, controlling for age and sex. The relationship between blood plasma concentrations and Glasgow Outcome Scale-Extended (GOSE) categories was analysed using logistic regression analyses, respectively, controlling for age and sex. Owing to established predictions from prior research, we did not correct for multiple comparisons in our analyses associating the RAVLT with blood biomarkers.^28,29^ Our previous studies consistently show specific associations between the RAVLT with brain health indicators in our older cohort. We ran additional exploratory secondary analyses to examine the association between the blood biomarkers and other measures of cognition.

Analyses examining ^18^F-NAV4694 and ^18^F-MK6240 group differences and associations with blood plasma concentration were conducted using FSL randomize, using 5000 permutations and with age and sex included as covariates.^44^ To mitigate potential confounds arising from focal lesions, lesion masks were generated for each TBI participant and applied in the non-parametric PET analysis (for lesion overlap image, see Supplementary Fig. 2). Group differences in white matter microstructure, and associations with blood plasma concentrations, were assessed using a linear regression controlling for age, sex and brain volume. An FDR of 5% was applied to analyses involving white matter microstructure to control for the number of comparisons. The relationships between brain age and blood plasma concentrations were evaluated using linear regressions controlling for age and sex. These were computed for the TBI group only. Group comparisons for brain age were not conducted here because they have been reported in a previous manuscript.^28^ Linear regression was used to compare verbal-episodic memory performance between groups and to examine associations with plasma blood concentrations. Regressions involving verbal memory were controlled for age, sex and premorbid IQ.

## Results

### Demographic and clinical characteristics of TBI and control participants

This study included 90 participants with TBI aged between 40 and 85 years with a history of a single moderate–severe TBI (73% male; mean = 58.2 years, SD = 11.6; Table 1), recruited into the study at a mean of 22 years post-injury at study enrolment (SD = 6.7, range = 10–33 years). The most common cause of injury was car accidents (57%), followed by being a pedestrian hit by a motor vehicle (16%), motorcycle (8%), falls (6%) and bicycle accidents (6%). Participants predominantly had severe TBI: 98% of participants had a PTA duration >1 day (mean = 30.3, range = 1–140 days), 64% had a GCS between 3 and 8, 87% had an abnormal CT scan at the time of injury, and 48% had a visible focal lesion on MRI at study inclusion. Despite a severe injury, 57% had a good recovery based on the GOSE (scores ranging from 7 to 8) at the time of clinical assessment.

**Table 1:** Demographic and clinical characteristics of TBI and control participants

Characteristic	TBI (*n* = 90)	Control (*n* = 32)	*P*-value
Age at assessment, years, mean (SD), range	58.20 (11.571), 40.01–85.63	57.94 (10.38), 41.95–87.97	0.911^a^
Sex, *n* (%)			0.408^b^
Female	24 (26.7)	11 (34.4)	
Male	66 (73.3)	21 (65.6)	
Education, years, mean (SD), range	12.51 (2.60), 1.00–18.00	13.25 (2.40), 8.00–18.00	0.162^a^
WTAR score FSIQ UK, mean (SD), range	100.16 (9.26), 74.00–116.00	102.00 (6.26), 85.00–110.00	0.299^a^
Time since injury, years, mean (SD), range	21.97 (6.72), 10.11–33.44	–	
Mechanism of injury, *n* (%)			
Car accident	51 (56.7)	–	
Pedestrian	14 (15.6)	–	
Motorcycle	7 (7.8)	–	
Bicycle accident	5 (5.6)	–	
Fall	5 (5.6)	–	
Work related accident	4 (4.4)	–	
Assault	2 (2.2)	–	
Horse	2 (2.2)	–	
PTA duration, days, mean (SD), range	30.250 (30.92), 1.00–140.00	–	
*N*-Miss	2		
PTA category, *n* (%)			
*N*-Miss	2	–	
<1	2 (2.3)	–	
1–7	22 (25.0)	–	
7–28	30 (34.1)	–	
>28	34 (38.6)	–	
GCS score, mean (SD), range	7.66 (4.28), 3.00–15.00		
*N*-Miss	13	–	
GCS category, *n* (%)			
*N*-Miss	13	–	
3–8	49 (63.6)	–	
9–12	11 (14.3)	–	
13–15	17 (22.1)	–	
GOSE category^c^, *n* (%)			
*N*-Miss	2	–	
Good recovery	50 (56.8)	–	
Moderate disability	36 (40.9)	–	
Severe disability	2 (2.3)	–	
Focal brain lesion on MRI, *n* (%)			
N-Miss	7	–	
No	43 (51.8)	–	
Yes	40 (48.2)	–	
Abnormal CT results, *n* (%)			
Normal	9 (10.0)	–	
Abnormal	78 (86.7)	–	
Unknown	3 (3.3)	–	

GCS = Glasgow Coma Score; GOSE = Glasgow Outcome Scale-Extended; *N*-Miss = number of missing values; PTA = post traumatic amnesia; WTAR = Wechsler Test of Adult Reading.

^a^Evaluated using an ANOVA.

^b^Evaluated using Pearson’s χ^2^ test.

^c^GOSE categories were collapsed for brevity. Lower and upper severe disability were categorized into severe disability. Lower and upper moderate disability were collapsed into moderate disability. Lower and upper good recovery were collapsed into good recovery.

Thirty-two control participants were included in the present study. Control and TBI participants had similar ages at assessment (*P* = 0.911), sex (*P* = 0.408), completed years of education (*P* = 0.162) and premorbid IQ as assessed using the Wechsler Test of Adult Reading (UK norms; *P* = 0.299).

### Blood plasma UCH-L1 and P-tau181 are elevated in chronic TBI

**Figure 1:**
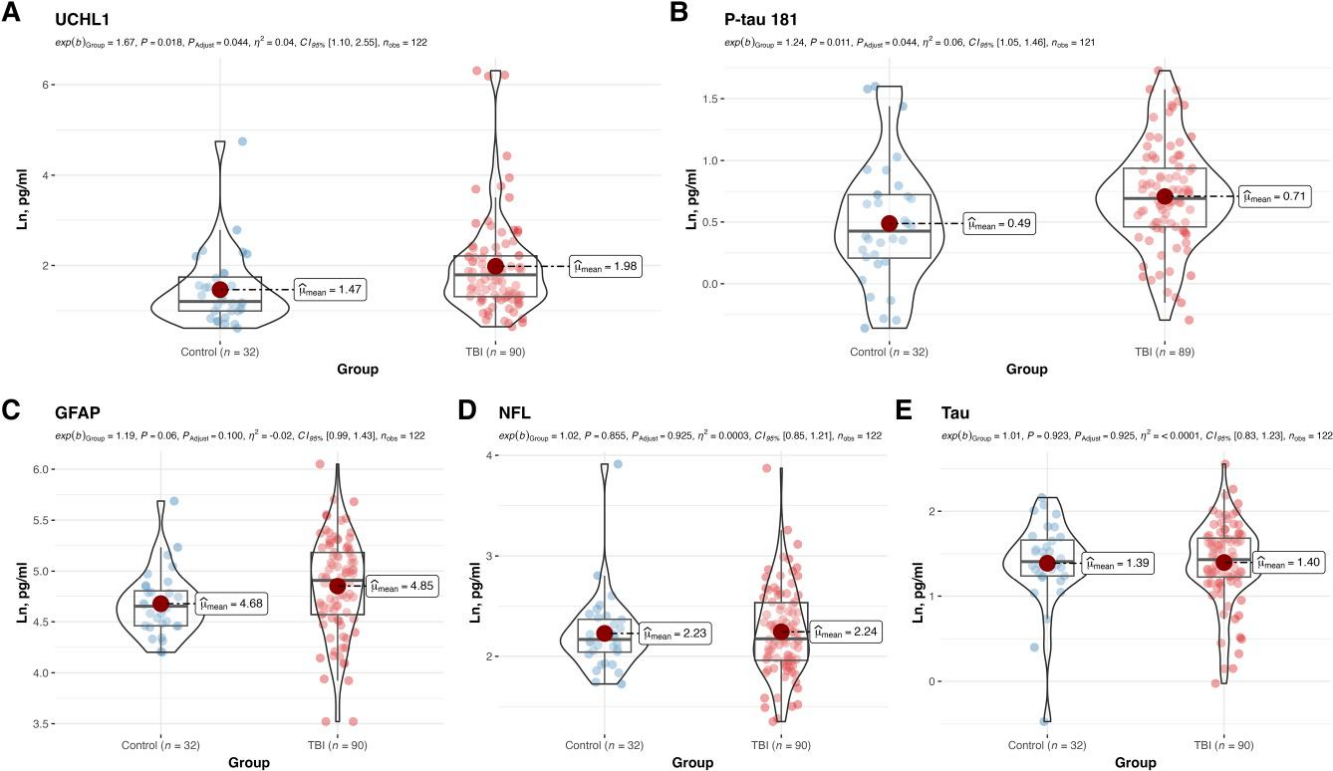
**Chronic traumatic brain injury is associated with elevated levels of UCH-L1 and phosphorylated tau.** TBI participants had elevated levels of ubiquitin C-terminal hydrolase L1 (UCH-L1; **A**) and phosphorylated tau (P-tau181; **B**), but not glial fibrillary acidic protein (GFAP; **C**), neurofilament light (NfL; **D**) and tau (**E**). The figure presents violin plots overlayed with a box plot. Individual participant values are included. Large red points indicate the group mean. Statistical information refers to the parameter coefficient for each blood marker, along with the corresponding *P*-value and 95% confidence intervals. *P*_Adjust_ values indicate *P*-values after FDR adjustment for multiple comparisons. exp = exponent; Ln = natural logarithm.

We compared the concentration of blood biomarkers between TBI and control participants (Fig. 1; for untransformed concentrations, see Supplementary Fig. 3), controlling for age and sex in regression analyses (for summary statistics, see Supplementary Table 1). TBI participants had elevated levels of UCH-L1 {exponent(*b*) = 1.67, *P* = 0.018, *P*_Adjust_ = 0.044, 95% CI [1.10, 2.55], AUC = 0.616; untransformed concentration values: *M*_TBI_ = 23.1, SD_TBI_ = 90.1, *M*_Control_ = 5.5, SD_Control_ = 10.8} and P-tau181 {exp(*b*) = 1.24, *P* = 0.011, *P*_Adjust_ = 0.044, 95% CI [1.05, 1.46], AUC = 0.632; untransformed concentration values: *M*_TBI_ = 2.6, SD_TBI_ = 4.0, *M*_Control_ = 1.9, SD_Control_ = 1.1}. The findings were still present after controlling for the history of medical conditions (for details, see Supplementary material, Methods 3) and APOE4 status (for details, see Supplementary material, Methods 4). There was no significant difference between groups in the concentration of GFAP, NfL or tau.

We investigated whether the duration of PTA and the GCS were correlated with blood plasma concentrations. Both PTA and GCS were included simultaneously in a linear regression model, with adjustments made for age and sex. Our analysis revealed a statistically significant positive association between PTA and concentrations of GFAP {*b* = 1.004, *P* = 0.049, 95% CI [1.00, 1.01]}, UCH-L1 {*b* = 1.012, *P* = 0.018, 95% CI [1.00, 1.02]} and P-tau181 {*b* = 1.004, *P* = 0.034, 95% CI [1.00, 1.01]} concentrations. However, there was no observed association between PTA and concentrations of NFL {*b* = 1.003, *P* = 0.167, 95% CI [1.00, 1.01]} or tau {*b* = 1.000, *P* = 0.892, 95% CI [1.00, 1.00]}. Furthermore, there was no significant association between GCS and any of the blood biomarkers (*P* > 0.05).

No statistically significant association was found between the time since injury and the concentration of blood biomarkers in the TBI cohort (*P* > 0.05). Additionally, we investigated whether blood biomarker concentration was linked to functional independence, assessed using the GOSE and categorized into ‘good recovery’ (scores of 7 or 8) and ‘disability’ (scores < 7) groups. Again, no statistically significant association was observed between functional independence and blood biomarker concentration in the TBI cohort (*P* > 0.05). Last, we examined whether the presence of focal lesions was associated with blood biomarker concentrations, controlling for age and sex. There was no statistically significant association between focal lesion and any of the blood biomarker concentrations (*P* > 0.05).

### Plasma biomarkers are unrelated to PET measures

We next examined whether blood biomarkers were associated with ^18^F-NAV4694 (amyloid-β) and ^18^F-MK6240 (tau neurofibrillary tangles) binding. No statistically significant difference in ^18^F-NAV4694 or ^18^F-MK6240 binding was found between the TBI and control participants. Moreover, concentrations of plasma biomarkers were not significantly associated with tracer binding for either PET measure, over the entire sample or within each group separately.

### White matter microstructure is disrupted in chronic TBI

White matter microstructure was compared between TBI and control participants (Supplementary Fig. 4). Overall, TBI participants displayed disrupted white matter microstructure across all white matter tracts for all microstructure metrics. The exception was log-FC, which was found generally to be preserved in TBI.

We examined the relationship between PET measures and white matter microstructure using the centiloid value as the primary measure summarizing the amyloid PET scan and quantified tau PET in four regions of interest: mesial temporal, meta-temporal, temporal–parietal and neocortex (Supplementary Fig. 5). After applying appropriate FDR correction for multiple comparisons, we found no statistically significant association between white matter microstructure and PET tracer retention (*P* > 0.05).

### Association between blood plasma biomarkers, white matter microstructure and brain age

We examined the association between the concentration of blood biomarkers, white matter microstructure and brain age. White matter microstructure was assessed using a range of measures, including more traditional diffusion-tensor imaging metrics, including FA, ADC, AD and RD. We also examined fixel-based measures, including FD and log-FC. The brain age gap (brain predicted − chronological age) was used as the primary measure for the association between brain age and blood plasma concentrations.

**Figure 2:**
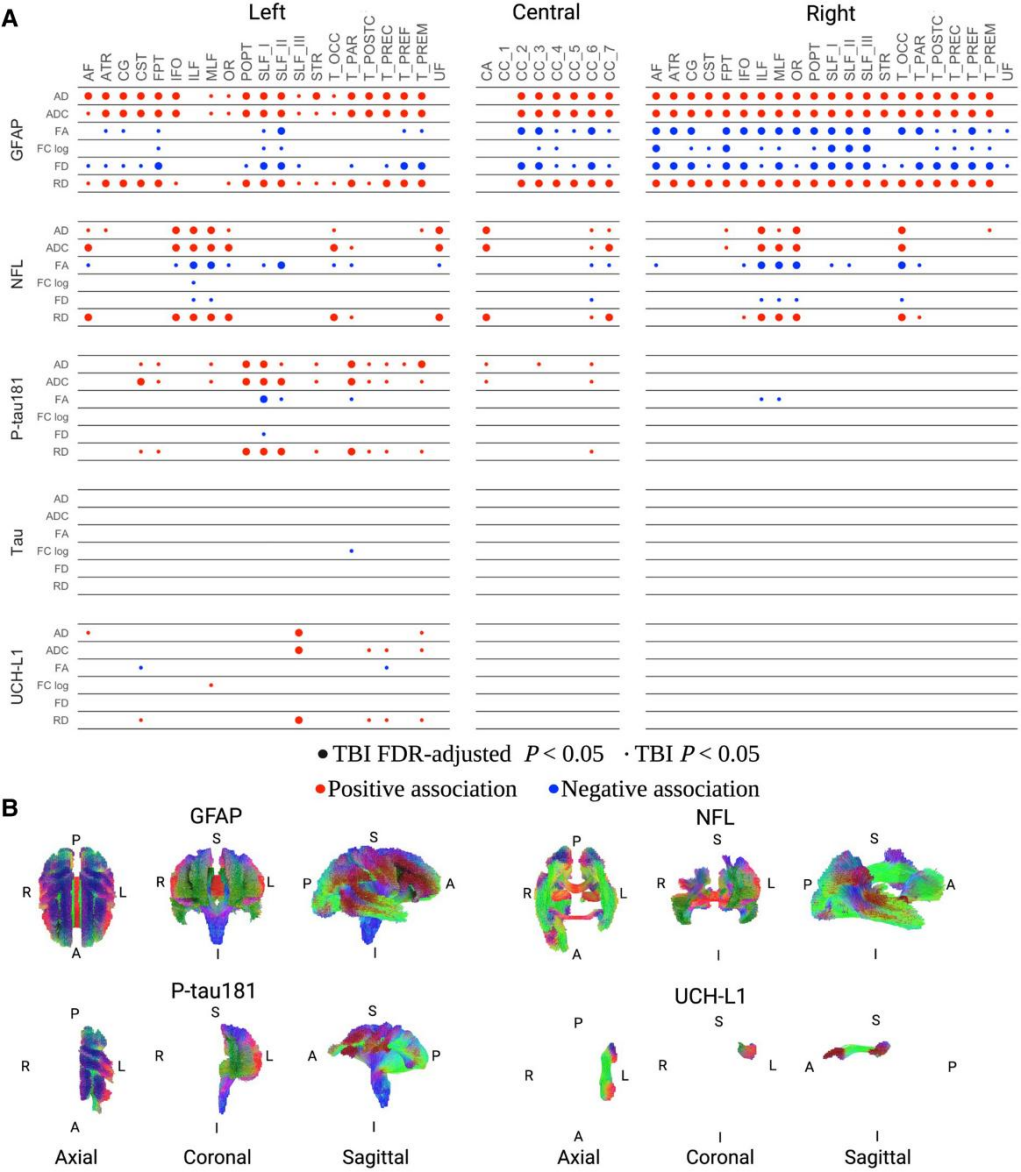
**Blood plasma is associated with white matter microstructure following traumatic brain injury.** This figure illustrates the regression results for each blood biomarker on measures of white matter microarchitecture in participants with traumatic brain injury (TBI), with adjustments made for age, sex and brain volume. (**A**) Both uncorrected *P*-values and false discovery rate (FDR)-corrected *P*-values are given. Large dots denote associations surviving FDR correction for multiple comparisons, while small dots represent associations statistically significant only using uncorrected *P*-values. Furthermore, red dots signify positive associations between white matter and blood measures, whereas blue dots indicate a negative association. Our findings reveal that higher concentrations of plasma glial fibrillary acidic protein (GFAP) exhibited the strongest association with various white matter measures, followed by neurofilament light (NfL), phosphorylated tau (P-tau181) and ubiquitin C-terminal hydrolase L1 (UCH-L1). Notably, blood concentration of tau showed no significant association with any white matter tract after FDR correction. (**B**) Visual representation of these results on the brain, with tract directionality indicated by colour. Only associations surviving FDR correction are presented in the axial, coronal and sagittal orientations. AF = arcuate fasciculus; ATR = anterior thalamic radiation; CA = commissure anterior; CC_1 = corpus callosum rostrum; CC_2 = corpus callosum genu; CC_3 = corpus callosum rostral body (premotor); CC_4 = corpus callosum anterior midbody (primary motor); CC_5 = corpus callosum posterior midbody (primary somatosensory); CC_6 = corpus callosum isthmus; CC_7 = corpus callosum splenium; CG = cingulum; CST = corticospinal tract; FPT = fronto-pontine tract; IFO = inferior occipital-frontal fascicle; ILF = inferior longitudinal fascicle; MLF = middle longitudinal fascicle; OR = optic radiation; POPT = parieto-occipital pontine; SLF_I = superior longitudinal fascicle I; SLF_II = superior longitudinal fascicle II; SLF_III = superior longitudinal fascicle III; T_OCC = thalamo-occipital; T_PAR = thalamo-parietal; T_POSTC = thalamo-postcentral; T_PREF = thalamo-prefrontal; T_PREM = thalamo-premotor; T_PREC = thalamo-precentral; UC = uncinate fascicle.

Statistically significant associations between blood plasma and white matter microstructure were present when only considering TBI participants (Fig. 2). GFAP was most strongly associated with poorer white matter microstructure, followed by NfL. Statistically significant associations between P-tau181, UCH-L1 and white matter microstructure were present only in the left hemisphere. No significant association was found between blood tau and white matter microarchitecture. No significant associations, surviving FDR correction, between blood plasma and white matter microstructure were found when limiting the analysis to the control group.

There was a statistically significant association between GFAP concentration and brain age gap for TBI participants {*b* = 1.02, *P* = 0.018, 95% CI [1.00, 1.03]}. There was no association between brain age gap and NfL {*b* = 1.01, *P* = 0.075, 95% CI [1.00, 1.02]}, tau {*b* = 1.01, *P* = 0.122, 95% CI [1.00, 1.02]}, UCH-L1 {*b* = 1.02, *P* = 0.189, 95% CI [0.99, 1.05]} or P-tau181 {*b* = 1.01, *P* = 0.079, 95% CI [1.00, 1.02]}. However, the association between GFAP and brain age gap did not survive FDR correction for multiple comparisons. No statistically significant associations between blood plasma and brain age gap were found when limiting the analysis to the control group.

### Verbal-episodic memory neuropsychological performance

**Figure 3:**
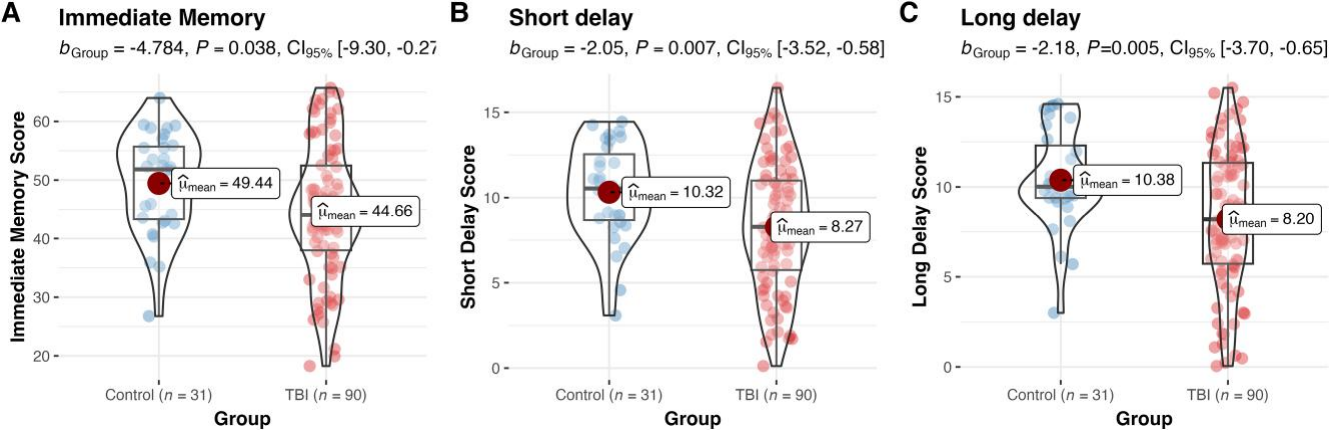
**Chronic single moderate–severe traumatic brain injury is associated with impaired verbal-episodic memory.** A single chronic moderate–severe traumatic brain injury (TBI) was found to be associated with impaired immediate (**A**), short delay (**B**) and long delay (**C**) verbal-episodic memory, in comparison to healthy controls. Group comparisons controlled for age at assessment, sex and premorbid IQ. Large red circles denote the group mean.

TBI and control participants were compared on verbal-episodic memory, controlling for age, sex and premorbid IQ (Fig. 3; for detailed analyses with clinical variables, see Supplementary material, Methods 4). TBI participants demonstrated impaired performance on all RAVLT measures, including immediate memory {for summary statistics, see Supplementary Table 2; *b*_ImmediateMemory_ = −4.78, SE = 2.28, *P* = 0.038, 95% CI [−9.30, −0.27]}, short delay {*b*_ShortDelay_ = −2.05, SE = 0.74, *P* = 0.007, 95% CI [−3.52, −0.58]} and long delay {*b*_LongDelay_ = −2.18, SE = 0.77, *P* = 0.005, 95% CI [−3.70, −0.65]}.

### Associations between plasma biomarkers and cognition

**Figure 4:**
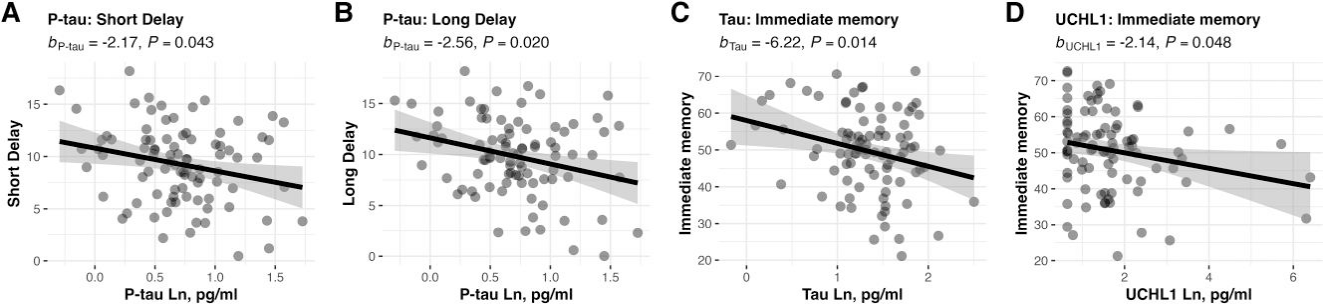
**Higher concentrations of phosphorylated tau, tau and UCH-L1 are associated with reduced verbal-episodic memory in chronic traumatic brain injury.** Following traumatic brain injury (TBI), higher blood concentration of phosphorylated tau (P-tau181) was associated with reduced verbal-episodic short delay (**A**) and long delay (**B**) recall. Higher concentrations of tau were associated with poorer immediate memory (**C**). Higher concentrations of ubiquitin C-terminal hydrolase L1 (UCH-L1) were associated with poorer immediate memory (**D**). Ln = natural logarithm.

Last, we examined the association between the blood biomarkers and verbal-episodic memory using regression, controlling for age, sex and premorbid IQ. For TBI participants, P-tau181 was associated with poorer short delay {Fig. 4A; *b*_P-tau_ = −2.17, SE = 1.06, *P* = 0.043, 95% CI [−4.28, −0.07]} and long delay {Fig. 4B; *b*_P-tau_ = −2.56, SE = 1.08, *P* = 0.020, 95% CI [−4.71, −0.41]}. In the TBI sample, tau was also associated with worse performance on immediate memory {Fig. 4C; *b*_Tau_ = −6.22, SE = 2.47, *P* = 0.014, 95% CI [−11.14, −1.30]}. Last, in TBI a higher concentration of UCH-L1 was associated with poorer immediate memory {Fig. 4D; *b*_UCH-L1_ = −2.14, SE = 1.07, *P* = 0.048, 95% CI [−4.26, −0.01]}.

We ran secondary exploratory analyses examining the relationship between blood biomarkers and our other cognitive measures. For TBI participants, a higher concentration of UCH-L1 was associated with poorer verbal fluency on the controlled oral word association test {*b*_UCH-L1_ = −2.36, SE = 1.00, *P* = 0.021, 95% CI [−4.35, −0.37]}, and a higher concentration of P-tau181 was associated with poorer performance on logical memory I {*b*_P-tau181_ = −2.61, SE = 1.17, *P* = 0.029, 95% CI [−4.94, −0.27]} and ROCF copy score {*b*_P-tau181_ = −2.68, SE = 1.27, *P* = 0.037, 95% CI [−5.20, −0.17]}.

## Discussion

In this study, we found that participants who had sustained a single moderate–severe TBI ≥10 years ago had elevated plasma P-tau181 and UCH-L1 levels in comparison to non-injured controls. ^18^F-MK6240 and ^18^F-NAV4694 binding were not elevated in TBI participants, nor was there a relationship between plasma biomarker concentration and binding for either PET measure or brain age. Plasma P-tau181, UCH-L1, GFAP and NfL were significantly associated with poorer white matter microstructure, and higher concentrations of P-tau181, UCH-L1 and tau were associated with worse verbal-episodic memory on neurocognitive testing following TBI.

Prior research has shown that the concentration of plasma P-tau181 is positively correlated with TBI severity and functional outcome in the acute and subacute period.^23^ To our knowledge, this is the first report of elevated plasma P-tau181 in the chronic period in participants who had experienced a remote single moderate–severe TBI. Plasma P-tau181 has been found to be a sensitive and specific marker of Alzheimer’s disease pathology, namely, Aβ and tau neurofibrillary tangles.^45-51^ In contrast, a recent study in the neurodegenerative condition amyotrophic lateral sclerosis cautions against plasma P-tau181 being used as a universal diagnostic screener that is specific for Alzheimer’s disease, finding that elevated levels of plasma P-tau181 were unrelated to Alzheimer’s disease pathology.^52^ Our negative findings on ^18^F-MK6240 and ^18^F-NAV4694 PET, coupled with the notably lower magnitude difference in P-tau181 levels between groups compared with those typically observed in Alzheimer’s disease, suggest that the ongoing neuropathological process detected in our sample diverges from the characteristic profile of Alzheimer’s disease, aligning with significant recent discoveries.^53^

Furthermore, clinical studies conducted in this group have not shown evidence of disproportionate cognitive decline following TBI and support the negative PET findings.^31,54^ However, in our sample, higher plasma P-tau181 concentrations were associated with poorer verbal-episodic memory. Baseline plasma P-tau181 concentrations have been found to be correlated with future tau PET uptake in temporoparietal regions, steeper declines in cognition, greater Aβ burden, and brain neurodegeneration/atrophy.^55,56^ Therefore, our findings require follow-up to inform the nature of the neuropathology that underpins the elevated plasma P-tau181 and to determine whether these baseline associations between plasma P-tau181 and verbal-episodic memory are predictive of future disease progression.

Our plasma P-tau181 findings are complemented by the results of elevated levels of plasma UCH-L1 following TBI. The ubiquitin–proteaseome system plays a vital role in targeting proteins for degradation. There is evidence that the aggregation of pathological proteins, common to neurodegenerative diseases, inhibits the activity of the ubiquitin–proteaseome system.^57^ Inhibition of the ubiquitin–proteaseome system itself can result in secondary pathological processes. Within the ubiquitin–proteaseome system, UCH-L1 is a deubiquitinizing enzyme that is abundant in the neuronal cytoplasm, where it plays an important role in the maintenance of axonal integrity, axonal transport and protein homeostasis.^58,59^ Upon neuronal stress or injury, UCH-L1 function can be dysregulated, is released from the neuron, and eventually reaches circulation.^60^ Elevated circulating levels of UCH-L1 have been found acutely after moderate–severe TBI, although, to our knowledge, our present findings are the first to show elevated levels in the chronic stages. One previous study examined serum UCH-L1 across the TBI severity spectrum for ≤5 years post-injury. Although there was a trend towards increased UCH-L1 in the severe TBI group, the data set was highly variable amongst the 25 participants.^27^

Our understanding of UCH-L1 levels in blood or CSF in human neurodegenerative diseases remains limited. Nonetheless, accumulating evidence indicates that conditions such as amyotrophic lateral sclerosis, Alzheimer’s disease and Parkinson’s disease are linked to altered levels of UCH-L1 in CSF, serum and plasma.^61-64^ This elevation might stem from various neurodegenerative mechanisms. It is plausible that ongoing neuronal damage characterizing these diseases contributes to elevated UCH-L1 levels. Additionally, compromised integrity of the blood–brain barrier might facilitate the leakage of proteins, including UCH-L1, from the brain into the circulation. A recent study by our team has suggested possible compromise within the neurovascular unit in this cohort.^29^ Moreover, disruptions in protein clearance pathways, particularly involving the ubiquitin–proteaseome system and autophagy, might result in the accumulation of misfolded or aggregated proteins within neurons. This build-up can trigger neuronal stress and damage, potentially leading to the extracellular release of UCH-L1 into the bloodstream.^60^

Our diffusion MRI findings identified widespread disruption of white matter microstructure in participants in the chronic phase post-TBI, and previous research has found this to be a predictor of later neurodegeneration in this group of patients.^65^ Plasma P-tau181, UCH-L1, GFAP and NfL levels were associated with white matter microstructure measures amongst the TBI participants, which is consistent with previous findings post-TBI and other neurodegenerative conditions.^25,27^ Although it is possible that the circulating biomarkers might be a direct reflection of pathophysiology contributing to white matter disruption, there are other explanations for these associations, and further studies into these relationships are required. For example, it could be that other mechanisms underlie or contribute to both our blood and MRI findings. Neuroinflammation and oxidative stress have both been linked to neuroaxonal injury, gliosis and proteopathies.^66^ Moreover, lack of elevated plasma NfL, GFAP and tau in our sample suggests that white matter changes might not be progressive in our sample, or indeed confounded by proteins stemming from the periphery rather than the brain.^67^ Once again, a longitudinal examination is required to ascertain whether the relationship between plasma markers and white matter microarchitecture is associated with subsequent white and grey matter neurodegeneration.

### Limitations

Despite being the largest biomarker study of its kind in subjects who have experienced a single remote moderate–severe TBI, our study nevertheless adopted a cross-sectional design. This design precludes analysis to demonstrate progressive neurodegenerative processes definitively. Future longitudinal examinations will allow us to assess the relationship between plasma biomarker concentrations and subsequent changes in cognition, brain Aβ and tau burden, and both white and grey matter brain atrophy neurodegeneration. We were able to rule out several potential confounding factors in our study, including history of medical disorders and APOE4 status. Nevertheless, there might be other confounding factors not measured in the present study. For example, presence of current renal dysfunction is known to influence concentrations of blood biomarkers. Furthermore, it is essential to incorporate newer blood-based biomarkers, such as brain-derived tau.^67^ These biomarkers exhibit higher specificity for distinct types of neurodegeneration, thereby offering enhanced precision in assessing long-term neuropathological changes following TBI. Finally, our plasma protocol deviated from conventional methods by subjecting samples to multiple rounds of centrifugation at varying speeds, aiming to enhance the quantification of Aβ. Although no evidence suggests that this procedure affects the quantification of other plasma proteins, additional studies using conventional protocols on independent samples are warranted for validation. Lastly, we urge other researchers to replicate these findings in independent cohorts with diverse mechanisms of injury and at various time points following their injury.

## Conclusions

Taken together, the findings of the present study suggest that plasma P-tau181 and UCH-L1 can provide biomarkers of ongoing neuropathology in the long-term, chronic phase, following a single moderate–severe TBI. However, longitudinal assessment is required to inform the clinical diagnostic and prognostic utility of these blood biomarkers.

## Supplementary Material

awae255_Supplementary_Data

## Data Availability

The data and scans from this study will be made available in de-identified format to researchers via the Federal Interagency Traumatic Brain Injury Research (FITBIR) database (https://fitbir.nih.gov/).
