# Supplementary material for: Plasma biomarkers in chronic single moderate–severe traumatic brain injury
Source: Brain. 2024 Sep 24;147(11):3690–701. doi: 10.1093/brain/awae255 (PMC11531850; doi:10.1093/brain/awae255)
Supplement: awae255_Supplementary_Data [file awae255_supplementary_data.pdf]

Supplementary material

Supplementary Methods 1. Summary table demonstrating result of participant matching procedure
Supplementary Methods 2. MRI acquisition and processing methods
Supplementary Methods 3. Relationship between history of health comorbidities and plasma markers
Supplementary Methods 4. Relationship between APOE4 and plasma markers
Supplementary Methods 5. Details verbal-episodic analyses
Supplementary Fig 1. Tractseg included tracts visualisation
Supplementary Fig 2. Lesion overlap map for TBI participants
Supplementary Fig 3. Blood marker group comparison using untransformed values
Supplementary Fig 4. White matter microstructure group comparison
Supplementary Fig 5. Association between white matter microstructure and PET measures
Supplementary Table 1. Summary statistics for plasma markers
Supplementary Table 2. Summary statistics for verbal-episodic measure

Supplementary Methods 1. Summary table demonstrating result of participant matching procedure

In previous publications, we found that TBI and control participants were not match with respect to age at assessment, sex, and premorbid IQ. In the current study, we performed an a priori matching procedure to ensure that differences in demographics did not bias any analyses between TBI and control participants with respect to biomarkers. The MatchIt package in r was used to match control participants to the TBI sample on age at assessment, sex, and premorbid IQ. ‘Cardinality matching’ was used within this package, which finds the largest matched set that satisfies the balance constraints between groups. Importantly, we chose a priori to include all TBI participants; instead, only control participants were excluded to ensure acceptable matching. We include the MatchIt command below, along with the output from the matching procedure. This table presents the means and standardised mean differences before and after matching as well as information regarding unmatched/discarded control participants.

**Matching procedure and output summary**			
**Command**			
Group ~ age_at_assessment + sex + wtar_fsiq_uk, data = dataset, method = "cardinality", ratio = NA, tols = 0.2			
**Summary of Balance for All Data:**			
	**Means - TBI**	**Means - Control**	**Std. Mean Diff**
Age at assessment	58.2011	60.2112	-0.1737
Sex – Female	0.2667	0.4211	-0.3491
Sex – Male	0.7333	0.5789	0.3491
WTAR FSIQ UK	100.1556	106.4035	-0.6748
**Summary of Balance for Matched Data:**			
	**Means - TBI**	**Means - Control**	**Std. Mean Diff**
Age at assessment	58.2011	57.9410	0.0225
Sex – Female	0.2667	0.3438	-0.1743
Sex – Male	0.7333	0.6562	0.1743
WTAR FSIQ UK	100.1556	102.0000	-0.1992
**Sample Sizes:**	**TBI**	**Control**	
All	90	57	
Matched	90	32	
Unmatched	0	25	
Discarded	0	0	

Ho, D. E., Imai, K., King, G., & Stuart, E. A. (2011). MatchIt: Nonparametric Preprocessing for Parametric Causal Inference. In *Journal of Statistical Software* (Vol. 42, Issue 8, pp. 1–28). https://doi.org/10.18637/jss.v042.i08

Supplementary Methods 2. MRI acquisition and processing methods

T1-weighted images were acquired with the following parameters: resolution = 1 x 1 x 1mm, repetition time = 2300ms, echo time = 3.65ms, field of view = 208 x 240 x 256mm, flip angle = 9°, number of slices = 208 slices, slice thickness = 1mm. A diffusion-weighted sequences was acquired with the following parameters: resolution = 2.5 x 2.5 x 2.5mm, 71 diffusion-weighted images ($b = 3{,}000$ s/mm^2) and one nondiffusion-weighted image, repetition time = 8400ms, echo time = 110ms, field of view = 150 x 240 x 240mm, number of axial slices = 59. A reverse-phase image was acquired for distortion correction.

Supplementary Methods 3. Relationship between history of health comorbidities and plasma markers

Plasma markers have been found to be associated with health comorbidities. We examined the association between medical history and plasma markers to ensure that health comorbidities did not confound group differences. Individuals were considered to have health comorbidities if they had a history of high blood pressure, high cholesterol, angina, atrial fibrillation, heart attack, stroke diabetes, cancer, thyroid disease, gastric complaints, arthritis, kidney disease, or liver disease. There were 62% of TBI participants and 48% of control participants with history of health comorbidity. There was no statistically significant difference between groups in the proportion of individuals reporting a health comorbidity ($\chi^2 = 1.768$, $p = 0.18$). History of health comorbidity was not associated with tau ($b_{HealthCoomrbid} = -0.02$, $SE = 0.098$, $p = 0.842$, $CI_{95\%}$ [-0.20, 0.16]), UCHL1 ($b_{HealthCoomrbid} = 0.22$, $SE = 0.20$, $p = 0.274$, $CI_{95\%}$ [-0.18, 0.62]), or P-tau181 ($b_{HealthCoomrbid} = 0.09$, $SE = 0.08$, $p = 0.284$, $CI_{95\%}$ [-0.07, 0.25]) concentrations. However, health comorbidities were associated with higher concentrations of NFL ($b_{HealthComorbid} = 0.45$, $SE = 0.10$, $p < 0.001$, $CI_{95\%}$ [0.26, 0.65]) and GFAP ($b_{HealthComorbid} = 0.20$, $SE = 0.10$, $p = 0.046$, $CI_{95\%}$ [0.00, 0.39]),

Supplementary Methods 4. Relationship between APOE4 and plasma markers

The APOE e4 allele status is known to be associated with plasma P-tau. We examine whether having at least one e4 haplotype versus none was more common in the TBI compared to the control group. This was to ensure that APOE status was not confounding our group difference with respect to concentrations of P-tau181. 19% of TBI participants and 26% of controls had at

least one e4 haplotype. There was no statistically significant difference between groups in the proportion of individuals with at least one e4 haplotype ($\chi^2 = 0.73$, $p = 0.39$). Including APOE e4 status into the regression model did not change the findings, nor was APOE e4 status statistically associated with P-tau181 plasma concentrations ($b_{APOE} = 0.03$, $SE = 0.10$, $p = 0.731$, $CI_{95\%}$ [-0.16, 0.23])

Supplementary Methods 5. Details verbal-episodic analyses

We examined the association between verbal-episodic memory and clinical variables using regression, controlling for age, sex, and premorbid IQ. Worse Immediate Memory was associated with greater duration of PTA ($b_{PTA} = -0.16$, $SE = 0.04$, $p < 0.001$, $CI_{95\%}$ [-0.23, -0.09]), lower GCS ($b_{GCS} = 1.08$, $SE = 0.31$, $p < 0.001$, $CI_{95\%}$ [0.46, 1.71]), and poorer functional outcomes ($b_{Good\ outcome - Moderate\ disability} = -6.76$, $SE = 3.11$, $p = 0.034$, $CI_{95\%}$ [-12.98, -0.54]; ($b_{Good\ outcome - Severe\ disability} = -22.52$, $SE = 7.62$, $p = 0.004$, $CI_{95\%}$ [-37.74, -7.31]). Poorer Short Delay was also associated with greater duration of PTA ($b_{PTA} = -0.04$, $SE = 0.01$, $p = 0.002$, $CI_{95\%}$ [-0.07, -0.02]), lower GCS ($b_{GCS} = 0.38$, $SE = 0.10$, $p < 0.001$, $CI_{95\%}$ [0.18, 0.59]), but only partially associated with functional outcomes ($b_{Good\ outcome - Moderate\ disability} = -1.75$, $SE = 1.01$, $p = 0.087$, $CI_{95\%}$ [-3.77, 0.26]; ($b_{Good\ outcome - Severe\ disability} = -7.61$, $SE = 2.47$, $p = 0.003$, $CI_{95\%}$ [-12.53, -2.68]). Lastly, worse Long Delay was also associated with greater duration of PTA ($b_{PTA} = -0.05$, $SE = 0.01$, $p < 0.001$, $CI_{95\%}$ [-0.07, -0.02]), lower GCS ($b_{GCS} = 0.35$, $SE = 0.11$, $p = 0.002$, $CI_{95\%}$ [0.13, 0.57]), but only partially associated with functional outcomes ($b_{Good\ outcome - Moderate\ disability} = -1.73$, $SE = 1.02$, $p = 0.093$, $CI_{95\%}$ [-3.76, 0.30]; ($b_{Good\ outcome - Severe\ disability} = -9.44$, $SE = 2.48$, $p < 0.001$, $CI_{95\%}$ [-14.41, -4.48]).

Supplementary Fig 1. Tractseg included tracts visualisation

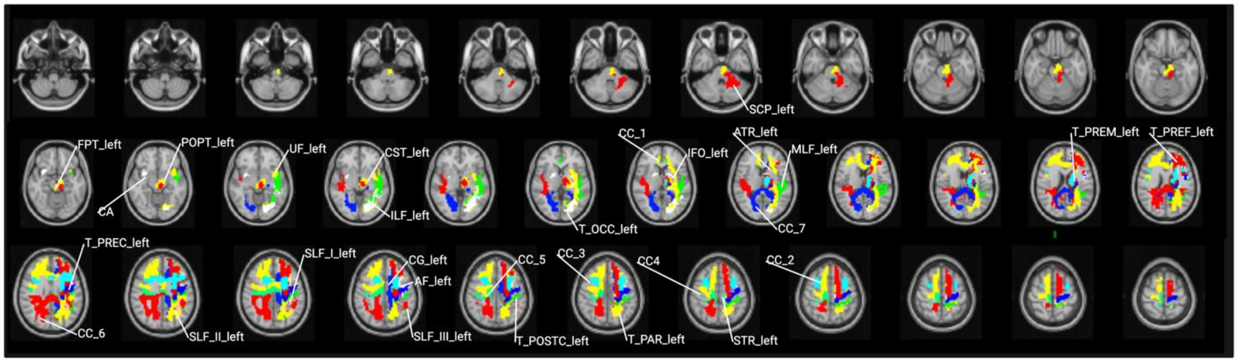

Visualisation showing the spatial layout of the TractSeg white matter tracts included in the current study, extract for each participant using. Only left tracts and tracts spanning both hemispheres ae presented. AF, Arcuate Fasciculus; ATR, Anterior Thalamic Radiation; CA, Commissure Anterior; CC_1, Corpus Callosum Rostrum; CC_2, Corpus Callosum Genu; CC_3, Corpus Callosum Rostral Body (Premotor); CC_4, Corpus Callosum Anterior Midbody (Primary Motor); CC_5, Corpus Callosum Posterior Midbody (Primary Somatosensory); CC_6, Corpus Callosum Isthmus; CC_7, Corpus Callosum Splenium; CG, Cingulum; CST, Corticospinal Tract; MLF, Middle Longitudinal Fascicle; FPT, Fronto-pontine Tract; IFO = Inferior Occipital-frontal Fascicle; ILF, Inferior Longitudinal Fascicle; OR, Optic Radiation; POPT, Parieto-occipital Pontine; SLF_I, Superior Longitudinal Fascicle I; SLF_II, Superior Longitudinal Fascicle II; SLF_III, Superior Longitudinal Fascicle III; UC, Uncinate Fascicle; T_PREF, Thalamo-prefrontal; T_PREM, Thalamo-premotor; T_PREC, Thalamo-precentral; T_POSTC, Thalamo-postcentral; T_PAR, Thalamo-parietal; T_OCC, Thalamo-occipital.

Supplementary Fig 2. Lesion overlap map for TBI participants

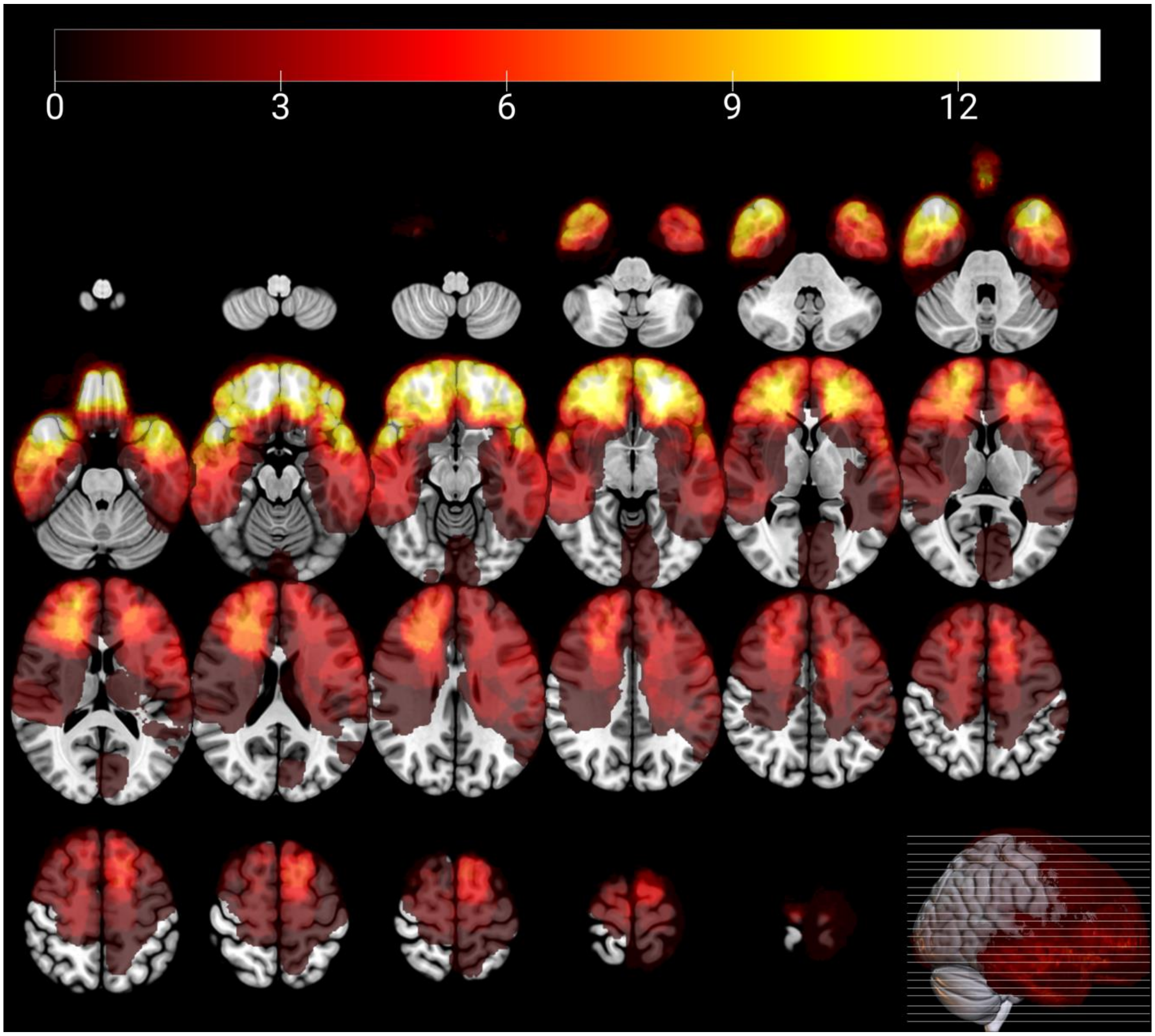

Overlap map of lesions segmented on T1-weighted images. Lesion masks were entered as voxelwise regressors for analyses examining PET measures. Colour bar indicates the number of participants with overlapping lesions for a given brain location.

Supplementary Fig 3. Blood marker group plots showing untransformed values. Nonparametric comparisons are conducted due to skewed distributions.

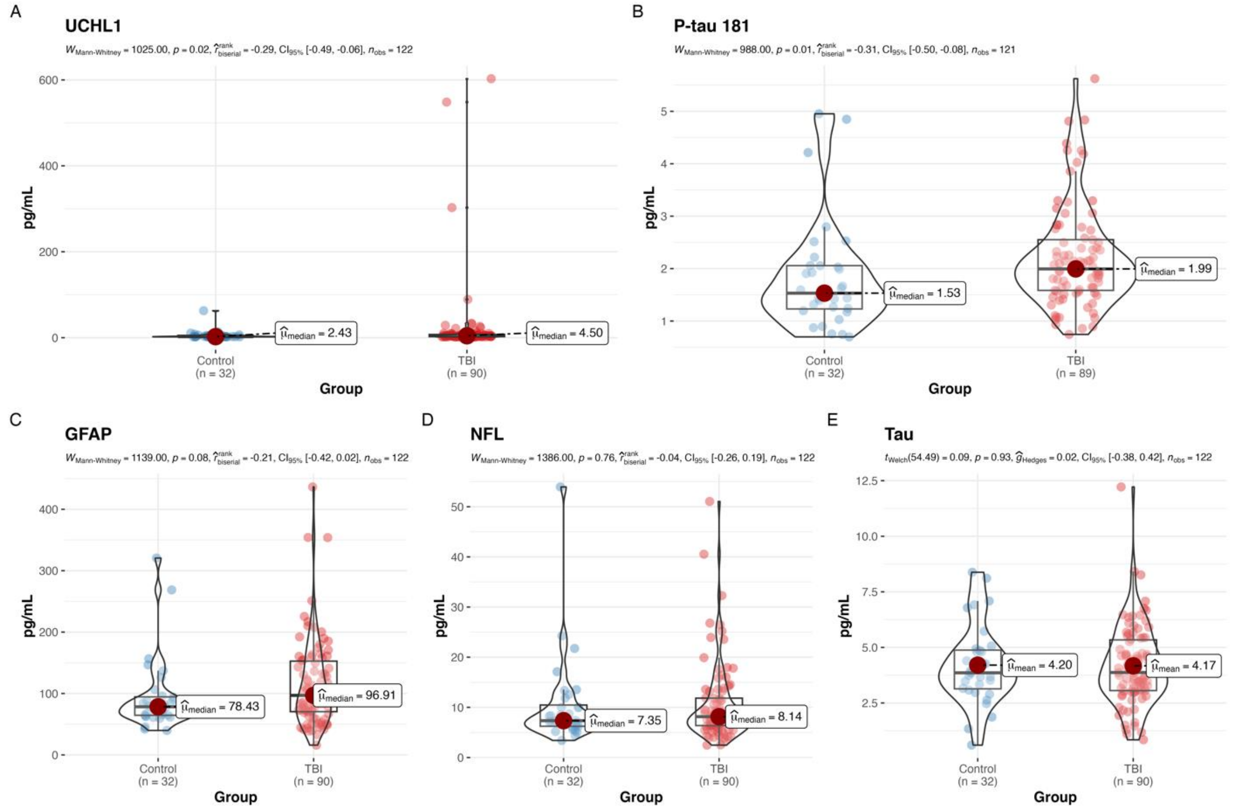

Supplementary Fig 4. White matter microstructure group comparison

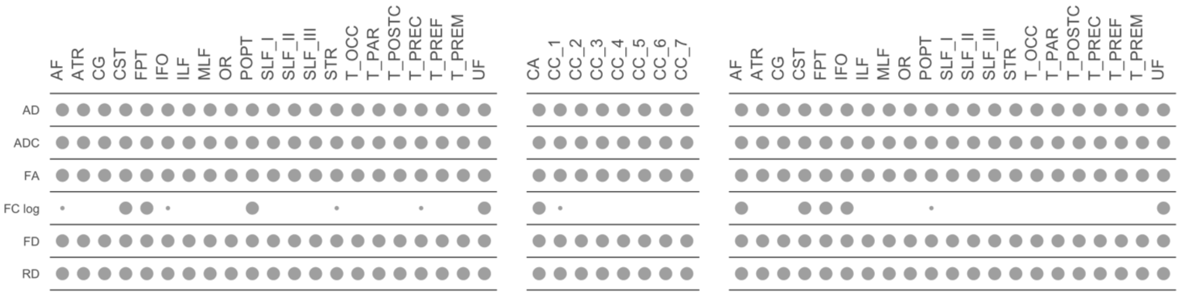

White matter microstructure was compared between TBI and control participants. TBI participants demonstrated widespread disruptions to white matter microarchitecture. Large circles indicate tracts surviving FDR correction whereas small circles indicated differences at $p < 0.05$, uncorrected.

Supplementary Fig 5. Association between white matter microstructure and PET measures

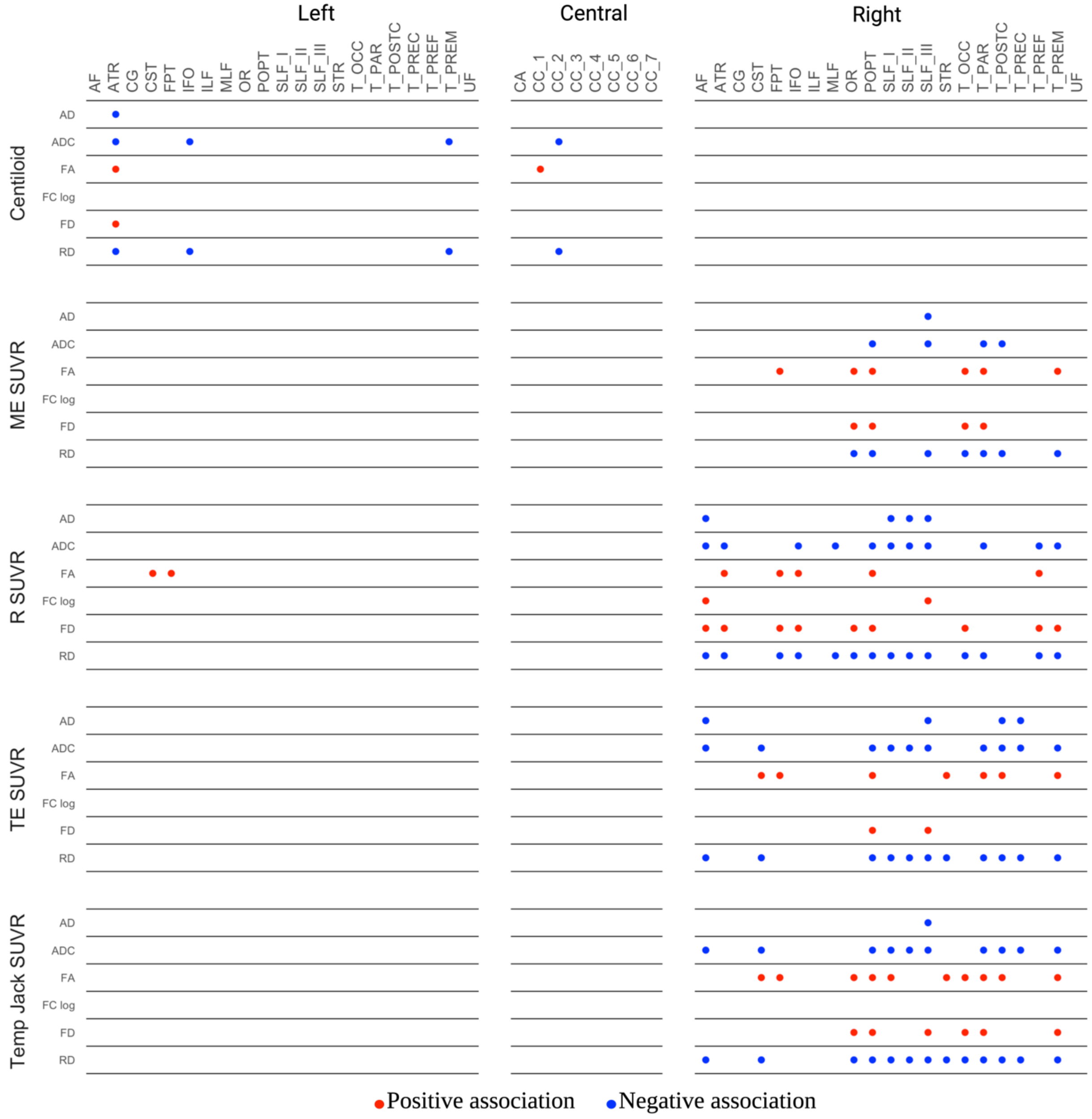

This figure illustrates the regression results for each PET measure on white matter microarchitecture metrics in TBI participants, with adjustments made for age, and sex. PET measures aligned with those used in a previously published paper focussing on PET in this cohort. Amyloid was quantified using Centiloid and tau was quantified in four ROIs using SUVR. The dots display associations uncorrected for multiple comparisons, but nevertheless meeting a p-value < 0.05. There were no significant associations once FDR correction was applied for multiple comparisons. Red dots signify positive associations between white matter and PET measures, whereas blue dots indicate a negative association. SUVR, Standardized Uptake Value Ratio; ME, mesial temporal region comprising entorhinal cortex, hippocampus, parahippocampus, and amygdala; R, region of interest ('rest of neocortex') comprising

dorsolateral and ventrolateral prefrontal, orbitofrontal cortex, gyrus rectus, superior temporal, and anterior cingulate; Temp Jack, meta-temporal region with some overlap with the regions of Me and Te comprising entorhinal cortex, hippocampus proper, parahippocampus, amygdala, fusiform, inferior and middle temporal gyri, temporo-occipital region and angular gyrus TE, region comprising inferior and middle temporal, fusiform, supramarginal and angular gyri, posterior cingulate/precuneus, superior and inferior parietal, and lateral occipital; AF, Arcuate Fasciculus; ATR, Anterior Thalamic Radiation; CA, Commissure Anterior; CC_1, Corpus Callosum Rostrum; CC_2, Corpus Callosum Genu; CC_3, Corpus Callosum Rostral Body (Premotor); CC_4, Corpus Callosum Anterior Midbody (Primary Motor); CC_5, Corpus Callosum Posterior Midbody (Primary Somatosensory); CC_6, Corpus Callosum Isthmus; CC_7, Corpus Callosum Splenium; CG, Cingulum; CST, Corticospinal Tract; MLF, Middle Longitudinal Fascicle; FPT, Fronto-pontine Tract; IFO = Inferior Occipital-frontal Fascicle; ILF, Inferior Longitudinal Fascicle; OR, Optic Radiation; POPT, Parieto-occipital Pontine; SLF_I, Superior Longitudinal Fascicle I; SLF_II, Superior Longitudinal Fascicle II; SLF_III, Superior Longitudinal Fascicle III; UC, Uncinate Fascicle; T_PREF, Thalamo-prefrontal; T_PREM, Thalamo-premotor; T_PREC, Thalamo-precentral; T_POSTC, Thalamo-postcentral; T_PAR, Thalamo-parietal; T_OCC, Thalamo-occipital

Supplementary Table 1. Summary statistics for plasma markers

	TBI		Control	
	**Ln Mean, SD**	**Raw Mean, SD**	**Ln Mean, SD**	**Raw Mean, SD**
UCHL1	1.7 (1.1)	23.0 (90.1)	1.2 (0.8)	5.5 (10.8)
P-tau181	0.7 (0.5)	2.6 (4.0)	0.5 (0.5)	1.9 (1.1)
GFAP	4.6 (0.6)	115.2 (72.0)	4.4 (0.5)	94.9 (59.7)
NFL	2.2 (0.6)	10.6 (7.9)	2.2 (0.6)	10.5 (9.2)
tau	1.3 (0.5)	4.2 (1.8)	1.3 (0.5)	4.2 (1.8)

Supplementary Table 2. Summary statistics for verbal-episodic measure

	TBI		Control	
	**Mean, SD** Age + Sex + Premorbid IQ Adjusted	**Raw Mean, SD**	**Mean, SD** Age + Sex + Premorbid IQ Adjusted	**Raw Mean, SD**
Immediate memory	44.7 (11.4)	44.4 (12.1)	49.4 (8.5)	50.3 (10.4)
Short delay	8.3 (3.7)	8.2 (3.8)	10.3 (2.8)	10.5 (3.1)

Long delay	8.2 (3.9)	8.1 (4.0)	10.4 (2.8)	10.5 (2.9)

This table presents the verbal-episodic summary statistics for TBI and control participants. We present the measures controlling for Age, Sex, and premorbid IQ. These are the summary statistics presented in Figure 3. The raw statistics are also presented.
